# Establishing Tetraploid Embryogenic Cell Lines of *Magnolia officinalis* to Facilitate Tetraploid Plantlet Production and Phenotyping

**DOI:** 10.3389/fpls.2022.900768

**Published:** 2022-05-04

**Authors:** Yanfen Gao, Junchao Ma, Jiaqi Chen, Qian Xu, Yanxia Jia, Hongying Chen, Weiqi Li, Liang Lin

**Affiliations:** ^1^Germplasm Bank of Wild Species, Kunming Institute of Botany, Chinese Academy of Sciences, Kunming, China; ^2^University of the Chinese Academy of Sciences, Beijing, China

**Keywords:** artificial polyploid, chromosome set doubling, embryogenic cell aggregate, colchicine, flow cytometry, *Magnolia officinalis*, somatic embryogenesis

## Abstract

The production of synthetic polyploids for plant breeding is compromised by high levels of mixoploids and low numbers of solid polyploid regenerants during *in vitro* induction. Somatic embryogenesis could potentially contribute to the maximization of solid polyploid production due to the single cell origin of regenerants. In the present study, a novel procedure for establishing homogeneous tetraploid embryogenic cell lines in *Magnolia officinalis* has been established. Embryogenic cell aggregate (ECA) about 100–200 μm across, and consisting of dozens of cells, regenerated into a single colony of new ECAs and somatic embryos following colchicine treatment. Histological analysis indicated that the few cells that survived some colchicine regimes still regenerated to form a colony. In some colonies, 100% tetraploid somatic embryos were obtained without mixoploid formation. New granular ECA from single colonies with 100% tetraploid somatic embryos were isolated and cultured individually to proliferate into cell lines. These cell lines were confirmed to be homogeneous tetraploid by flow cytometry. Many tetraploid somatic embryos and plantlets were differentiated from these cell lines and the stability of ploidy level through the somatic embryogenesis process was confirmed by flow cytometry and chromosome counting. The establishment of homogeneous polyploid cell lines, which were presumed to represent individual polyploidization events, might expand the phenotypic variations of the same duplicated genome and create novel breeding opportunities using newly generated polyploid plantlets.

## Introduction

Artificial polyploid induction can be used for the improvement of important plant traits, in support of crop breeding strategies (Dhooghe et al., 2011; Chen et al., 2020; Niazian and Nalousi, 2020; Touchell et al., 2020). It has been widely used in the breeding of fruit trees (Zeng et al., 2006; Dutt et al., 2010), ornamental plants (Lucía et al., 2015; Tu et al., 2018), and medicinal plants (Banyai et al., 2010; Chung et al., 2017; Chen et al., 2018). Whole genome duplication not only increases copies of existing genes but also produces genomic alterations, such as modulated gene expression and epigenetic changes that lead to various phenotypic variations (Chen et al., 2020; Niazian and Nalousi, 2020). Polyploid plants often exhibit better agronomic characteristics related to enhanced biomass, increased yield and tolerance to biotic and abiotic stresses (Niazian and Nalousi, 2020; Ruiz Valdés et al., 2020; Touchell et al., 2020).

*In vitro* regeneration systems provide a powerful tool for mitotic ploidy manipulation (Dhooghe et al., 2011; Touchell et al., 2020). The past two decades have seen a significant increase in the use of *in vitro* polyploid induction for a diverse range of taxa (Dhooghe et al., 2011; Niazian and Nalousi, 2020; Ruiz Valdés et al., 2020; Touchell et al., 2020). *In vitro* polyploid induction can be achieved by treating proliferative tissue with antimitotic agents followed by recovery of plantlets and screening of polyploid regenerants (Niazian and Nalousi, 2020). The success of *in vitro* polyploid induction is maximized when coupled with the development of efficient *in vitro* regeneration protocols (Touchell et al., 2020).

*In vitro* regeneration from preexisting meristems, such as apical meristems and nodal sections, has been predominantly used for polyploid induction of many species (Allum et al., 2007; Lucía et al., 2015; Fernando et al., 2019; Parsons et al., 2019). To obtain homogeneous polyploids using preexisting meristems, all initial cells within the three histogenic layers of the meristem need to be successfully affected. Otherwise, mixoploids or cytochimeras form, resulting in the need for serial tissue culture cycles for purification (Roux et al., 2001; Allum et al., 2007; Silva et al., 2019). The main bottlenecks of *in vitro* polyploid induction, such as low rates of solid polyploid initiation, high levels of mixoploid and low numbers of polyploid regenerants, are inherently correlated with the regeneration systems utilizing enhanced axillary shoot proliferation from preexisting meristems (Dhooghe et al., 2011; Touchell et al., 2020; Venial et al., 2020).

Somatic embryogenesis is a better regeneration system for *in vitro* polyploid induction than using preexisting meristems (Wu and Mooney, 2002; Zeng et al., 2006; Acanda et al., 2015). The single cell origin of somatic embryos from embryogenic tissues allows solid polyploid formation while eliminating the occurrence of mixoploids (Acanda et al., 2015; Sanglard et al., 2017; Venial et al., 2020). In polyploid induction based on the somatic embryogenesis system, however, the recovery of polyploid somatic embryos and plantlets is still impeded by necrosis and a reduced differentiation capacity of embryogenic tissues due to antimitotic agent toxicity (Zeng et al., 2006; Acanda et al., 2015). A decline in the relative proportion of polyploid cells in the mixed population following antimitotic agent treatment is due to the persistent lethality of the antimitotic agent and/or inferior growth ability of polyploid cells compared to diploid cells (Zeng et al., 2006). As a consequence, the proportion of polyploid somatic embryos decreases or is eventually lost during the prolonged regrowth and differentiation recovery phase post-treatment with antimitotic agents. In addition, toxicity of antimitotic agents affects the morphological development of somatic embryos and reduces their conversion into plantlets (Zeng et al., 2006; Venial et al., 2020). The induction and purification of polyploid cell lines is expected to eliminate the toxicity effect of antimitotic agents and facilitate subsequent polyploid production.

Plant embryogenic tissues can either proliferate as cell masses or differentiate into somatic embryos, depending on the presence or absence of adequate auxin in the medium (Nic-Can and Loyola-Vargas, 2016). Embryogenic tissues, following antimitotic agent treatment, usually regenerate during regrowth into a mixture of somatic embryos and new Embryogenic cell aggregates (ECAs) (Sanglard et al., 2017; Venial et al., 2020). It is clear that polyploid somatic embryos originate from cells with a duplicated chromosome set. New ECA formation can also originate from cells with a duplicated chromosome set. It is expected that polyploid cell lines could be purified and established by culturing small granular ECAs, which regenerate from embryogenic tissues following antimitotic agent treatment. This can be achieved using a medium that promotes cell proliferation, followed by screening with flow cytometry. Significant phenotypic variations between sibling polyploid clones are known to result from genetic and epigenetic changes occurring during individual polyploidization events (Podwyszyńska et al., 2021; Wójcik et al., 2022). The establishment different polyploid cell lines from the same diploid line, which are presumed to represent individual polyploidization events, might expand the phenotypic variations and create novel breeding opportunities.

Attaining a high proportion of polyploid cells in the mixed cell population following antimitotic agent treatment is a prerequisite for the purification of polyploid cell lines. This can only be achieved by use of very small ECAs instead of large tissues. In addition, synchronization of the tissue culture process before antimitotic agent treatment is also required because polyploidization is integrated with *in vitro* regeneration system (Dhooghe et al., 2011). Synchronization of somatic embryogenesis can be achieved by fractionation of the initial heterogeneous cell population, followed by the transfer of homogeneous cell clusters to a differentiation medium. In carrot, synchronized somatic embryo formation from cell clusters 31–47 μm across, and composed of 3–10 cells, reached more than 90% (Fujimura and Komamine, 1979; Osuga and Komamine, 1994). Size fractionation and plating of proembryonic masses (PEM) 38–140 μm in size on filter paper was key to mass production of synchronized and singularized yellow poplar somatic embryos (Merkle et al., 1990; Dai et al., 2004). For polyploid induction, the use of small homogeneous ECAs can allow the quick and uniform permeation of antimitotic agents, thus maximizing the proportion of affected cells. The cytotoxic effect of antimitotic agents can lead to dose dependent mortality of cells in explants. When small ECAs composed of dozens of cells are treated with antimitotic agents, the number of surviving cells can be very limited. The regeneration of somatic embryos and new ECAs from a very limited number of cells following antimitotic agent treatment could greatly facilitate the purification and establishment of polyploid cell lines.

*Magnolia officinalis* is a large tree found in the broad-leaved forests in central China. The cortex of *M. officinalis*, known as “Houpo,” has been used historically in Traditional Chinese Medicine (Luo et al., 2019). The main constituents are lignans, alkaloids, and volatile oils, among them the main active compounds are magnolol and honokiol (Lee et al., 2011; Yin et al., 2021). The active components of *M. officinalis* are also widely used in the cosmetics industry. *M. officinalis* is widely cultivated in China to supply cortex to the commercial market, but the plants need about 15 years of growth before production is commercially viable (Luo et al., 2019). The production of synthetic polyploids constitutes a novel opportunity to accelerate the breeding of *M. officinalis* and increase the production of active components in the cortex.

The objective of the present study was to explore an efficient procedure for establishing homogenous tetraploid embryogenic cell lines in *M. officinalis*. Tetraploid cell lines were successfully established and confirmed. The stability of ploidy level through a subsequent somatic embryogenesis process was evaluated in somatic embryos and plantlets differentiated from tetraploid cell lines. In addition, morphological changes of tetraploid somatic embryos and plantlets were assessed in relation to their diploid counterparts. This research shows the possibility of obtaining polyploid cell lines to overcome the bottlenecks of *in vitro* polyploid induction.

## Materials and Methods

### Initiation and Maintenance of Embryogenic Cultures

Proembryonic masses of *M. officinalis* were initiated from mature zygotic embryos in M1 medium (Table 1). Seeds were surface sterilized for 10 min in a 0.5% sodium dichloroisocyanurate solution containing a drop of Tween 20 and rinsed three times with sterile deionized water. Zygotic embryos were extracted from these seeds and inoculated on M1 medium (Table 1) in 9-cm diameter Petri dishes. M1 medium contained woody plant medium (McCown and Lloyd, 1981) with plant growth regulators previously used for the induction of PEMs in *Liriodendron tulipifera* (Merkle et al., 1990). The cultures were maintained in darkness at 25°C and subcultured monthly until PEMs were produced. The PEMs were then maintained on M2 medium (Table 1) and subcultured regularly at 2 weeks intervals.

**TABLE 1:** Tissue culture media used for embryogenic culture induction (M1), maintenance (M2), colchicine treatment (M3), and somatic embryo initiation (M4) of *Magnolia officinalis*.

	Medium			
Components	M1	M2	M3	M4
Casein hydrolysate	1 g L^–1^	1 g L^–1^	–	–
2, 4-D	1 mg L^–1^	1 mg L^–1^	–	–
6-BA	0.25 mg L^–1^	–	–	–
Phytagel	3 g L^–1^	3 g L^–1^	–	3 g L^–1^
Activated charcoal	1 g L^–1^	1 g L^–1^	–	1 g L^–1^
PVP	1 g L^–1^	1 g L^–1^	–	–

*All media contained Lloyd & McCown’s Woody plant medium with vitamins and supplemented with 30 g L^–1^ sucrose. 2,4-D, 2,4-dichlorophenoxyacetic acid; 6-BA, 6-Benzylaminopurine; PVP, polyvinylpyrrolidone (MW 40, 000).*

### Preparation of Embryogenic Cell Aggregates and Colchicine Treatment

**FIGURE 1:**
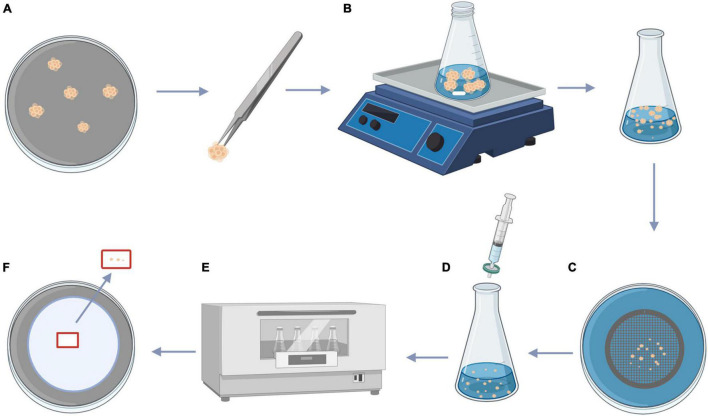
Procedure for size fractionation of ECAs and colchicine treatment. **(A)** PEMs of *Magnolia officinalis* proliferated on M2 medium. **(B)** PEMs of *M. officinalis* were transferred to 50 mL Erlenmeyer flask containing 20 mL of M3 liquid medium with the magnetic agitator, the Erlenmeyer flask was placed in a magnetic mixer at 0°C and 1,000 rpm for dispersion. **(C)** After 10 min, the dispersed PEMs were passed through 100 and 200 μm sieves, producing a homogeneous suspension composed of ECAs with a diameter of 100–200 μm. **(D)** Filter sterilized colchicine solution was added to the ECA suspensions. **(E)** ECAs were treated with colchicine at different concentrations on an orbital shaker (100 rpm) at 25°C for 24, 48, and 72 h. **(F)** Following colchicine treatment, ECAs were washed and plated on M4 medium for somatic embryo initiation. This figure was created using BioRender (https://biorender.com/).

After 14 days culturing on fresh M2 medium (Table 1), the actively dividing PEMs (Figure 1A) were used to obtain homogeneous ECAs with a diameter of 100–200 μm. PEMs (500 mg, fresh weight) were transferred to 50 mL Erlenmeyer flasks containing 20 mL of M3 liquid medium (Table 1) with a sterile magnetic agitator (size 7 mm × 30 mm). Then the Erlenmeyer flask was placed in an IKA C-MAG HS7 magnetic mixer at 0°C and 1,000 rpm for dispersion (Figure 1B). After 10 min, ECAs with a diameter of 100–200 μm were obtained by sieving with screens with 100 and 200 μm pores (Figure 1C).

Colchicine stock solution was filter-sterilized and added to 25 mL Erlenmeyer flask containing 5 mL M3 liquid medium with ECAs to reach final colchicine concentrations (w/v) of 0, 0.05, 0.1, 0.15, and 0.2% (Figure 1D). The cultures were incubated on a tube rotator mixer (120 rpm) at 25°C for 24, 48, and 72 h (Figure 1E). Following colchicine treatment, the ECAs were rinsed three times with sterilized M3 liquid medium, 1 mL of ECAs were then plated on a layer of sterilized filter paper. The filter paper with ECAs were then placed on top of semi-solid M4 medium and maintained at 25°C under a 16 h photoperiod (Figure 1F).

To estimate the number of ECAs in the cell suspension, aliquots of 1 mL of cell suspension were precipitated by centrifugation, and resuspended in 0.1 mL of M3 medium and pipetted onto a glass slide. Images were taken under an OLYMPUS SZX16 microscope and the number of ECAs were counted with CellSens Dimension software.

### Regeneration Following Colchicine Treatment

**FIGURE 2:**
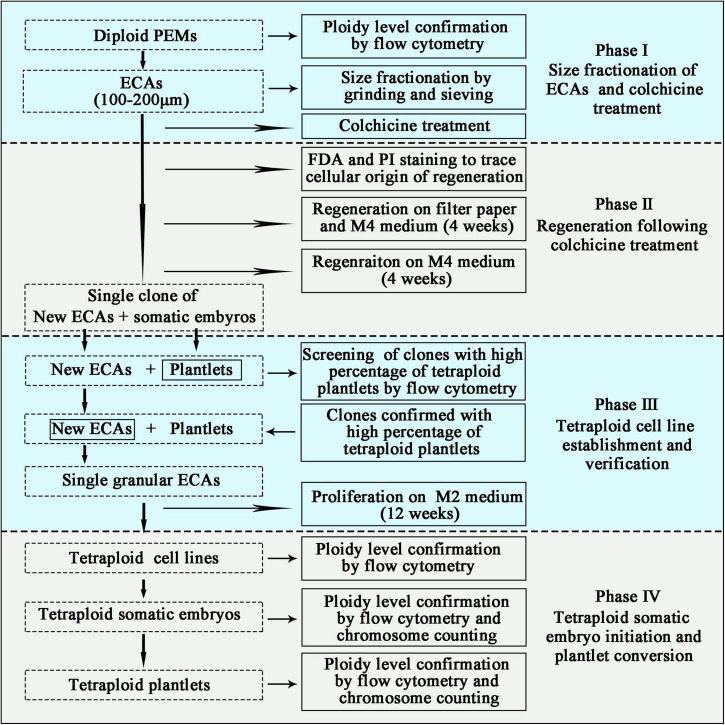
Schematic pipeline for the establishment of tetraploid cell lines of *Magnolia officinalis*. Sequential stages of colchicine treatment, regeneration, tetraploid cell line establishment and ploidy level verification are presented.

After plating the colchicine treated ECAs onto a piece of filter paper and cultured on semi-solid M4 medium for 4 weeks, each surviving ECA regenerated into a single colony consisting of somatic embryos and/or new ECAs (Figure 2). The number of somatic embryos produced per ECA was determined to evaluate the embryogenic potential of colchicine treated ECAs. Each colony was then picked up and cultured on semi-solid M4 medium without filter paper to enhance new ECA proliferation. Many granular ECAs appeared on most of colonies after 4 weeks (Figure 2).

For plantlet conversion, the cotyledonary somatic embryos were first transferred to 9 cm Petri dishes containing 20 mL of semi-solid M4 medium for germination. The cultures were maintained at 25°C under a 16 h photoperiod. The germinated somatic embryos were transferred to glass culture vessels containing 100 mL of semi-solid M4 medium and cultured at 25°C under a 16 h photoperiod.

### Fluorescein Diacetate and Propidium Iodide Staining

To trace the cellular origin of ECAs following colchicine treatment, fluorescein diacetate (FDA) and propidium iodide (PI) were used to label living cells and dead cells, respectively (Figure 2). ECAs were double stained immediately following grinding and sieving to assess the effect of the preparation process on the viability of cells. ECAs treated with colchicine (0.2% for 72 h) were double stained after 72 h of regeneration to indicate the position of surviving cells in the ECA, and to roughly evaluate the proportion of living cells. To stain the ECAs, 5 μL of 10 mg/mL FDA and 5 μL of 1 mg/mL PI were added to 1 mL of ECAs suspension, then incubated in the dark for 5 min. The working concentration of FDA and PI was 50 and 5 μg/mL, respectively. The ECAs were washed three times with deionized water and then observed with a 60 × oil objective by Confocal Laser Scanning Microscopy using an Olympus FV 1,000 system equipped with argon as an excitation source. FDA fluorescence was excited at 488 nm and collected with a 520–550 nm filter, PI was excited at 545 nm and collected with a 560–600 nm filter.

### Flow Cytometry Analysis

Flow cytometry was used to determine the ploidy level of cell lines, somatic embryos and plantlets (Figure 2). For DNA content determination, a small amount of plant tissue (typically 20 mg) was placed in 1 mL ice-cold nuclei isolation buffer (WPB buffer) in a Petri dish. Using a new razor blade, the tissue was immediately chopped in the buffer, and the homogenate filtered through a 42-μm nylon mesh into a labeled sample tube. The samples were incubated with the DNA fluorochrome PI for 30 min and the relative fluorescence of the stained nuclei was then measured. The cytometer was equipped with an argon ion laser operating at 488 nm. The PI fluorescence was collected by 600 nm fluorescence-2 (FL2) filter. Parameters for data acquisition were kept constant for all samples. The results acquired were later analyzed using Cell Quest software. The average coefficient of variation values (CV) for G1 peaks were used to evaluate the results. The results with CV < 5% were considered as reliable. Leaves of 24 plantlets from each of the colchicine treatments were collected to determine the ploidy level.

### Chromosome Counting

The ploidy level of tetraploid plantlets and somatic embryos verified by flow cytometry were further confirmed by chromosome counting to precisely determine the number of chromosomes (Figure 2). Root tips and globular somatic embryos were collected and incubated in 2 mM 8-hydroxyquinoline solution for 4 h at 25°C. Subsequently, the samples were washed three times with deionized water for 5 min each, and fixed in Carnoy’ s solution (ethanol: glacial acetic acid = 3:1) for 24 h at 4°C. After three washes with deionized water for 5 min each, the samples were digested for 8 min at 60°C in a solution of 45% acetic acid: 1 M HCl at 1:1. After three rinses with deionized water, the digested samples were squashed in a carbol-fuchsin solution for 15 min and placed on a slide. The cells were observed and imaged using a LEICA DM1000 microscope under a 100 × oil immersion objective lens.

### Stomata Characteristics

To compare the difference of stomata characteristics between diploid and tetraploid plantlets, nail polish imprints were made from the abaxial surface of leaves. Briefly, the abaxial surface of the leaves were covered with a thin layer of nail polish and allowed to dry. The polish imprints were then carefully lifted off with forceps and placed on a glass slide. The stomata were observed and imaged under LEICA DM5500 B microscope with LAS software. For evaluating the stomatal density, the number of stomata per field of view under the 40× objective was recorded in eight different images. The size of the image was measured to calculate the number of stomata per mm^2^. For stomatal length and width measurements, three stomata in each image were randomly selected and measured (*n* = 24).

### Establishment and Verification of Tetraploids Embryogenic Cell Lines

Flow cytometry were used for screening of clones with a high percentage of tetraploid plantlets. To establish tetraploid cell line production, single granular ECAs from the colonies confirmed with a high percentage of tetraploid plantlets were isolated and cultured individually on semi-solid M2 medium for proliferation (Figure 2). These cultures were maintained at 25°C in the dark and subcultured on new semi-solid M2 medium every 4 weeks. At the end of the third subculture, each single ECA proliferated into a single-ECA-derived cell line. Flow cytometry was then used to analyze the ploidy level of each single-ECA-derived cell lines (Figure 2).

### Phenotypic Analyses

To assess the effects of polyploidization, the size of the somatic embryos and biomass of the plantlets (3 months after somatic embryo germination) regenerated from diploid and tetraploid cell lines were measured and compared. Diploid and tetraploid cell lines were used to produce somatic embryos with the same method. ECAs with a diameter of 200–450 μm were obtained and plated on a piece of filter paper and cultured on semi-solid M4 medium for somatic embryo initiation. Thirty somatic embryos initiated from diploid and tetraploid cell lines were collected 5 weeks after somatic embryo initiation. Images were taken under an OLYMPUS SZX16 microscope. The length and diameter of somatic embryos were calculated with CellSens Dimension software. For plant conversion, germinated somatic embryos were transferred to glass culture vessels containing 100 mL of semi-solid M4 medium and cultured at 25°C under a 16 h photoperiod. Following 7 weeks culture, 12 *in vitro* plantlets of diploid and tetraploid status were collected, and the biomass was measured.

### Statistical Analysis

The colchicine treatment experiment was designed with a randomized complete block design, and each treatment contained five replicates. Data were processed and analyzed with SPSS 19.0 software using Duncan’s multiple range tests at *p* < 0.05. The measurement of somatic embryos, *in vitro* plantlets and stomata characteristics were analyzed with Student’s *t*-test at *p* < 0.05.

## Results

### Synchronization of the Somatic Embryogenesis Process

**FIGURE 3:**
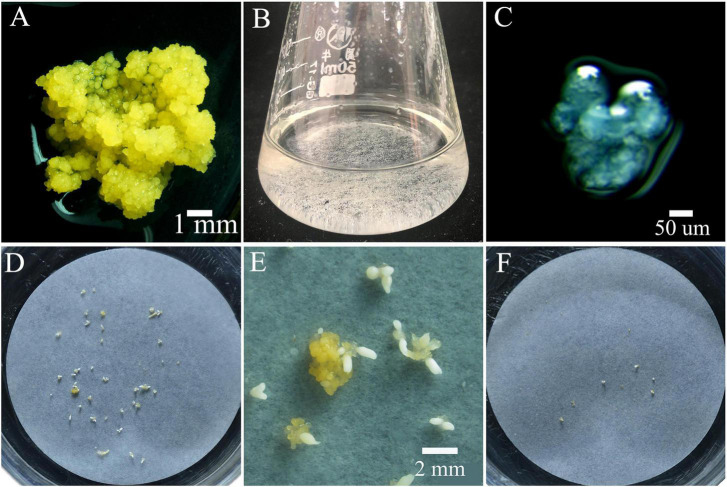
Proembryonic masses (PEMs) and ECAs of *Magnolia officinalis* used for polyploid induction by colchicine. **(A)** PEMs of *M. officinalis* 14 days after subculture. **(B)** ECAs with a diameter of 100–200 μm suspended in M3 liquid medium in a 50 mL Erlenmeyer flask. **(C)** Microscopic observation of the ECAs with a diameter of 100–200 μm. **(D)** Somatic embryo differentiation from ECAs plated on a piece of filter paper after 4 weeks culture on M4 medium. **(E)** A magnified view of somatic embryo differentiation from ECAs plated on a piece of filter paper on M4 medium. **(F)** Regrowth and somatic embryo differentiation of ECAs treated with colchicine, 4 weeks after colchicine treatments.

The PEMs of *M. officinalis* were initiated from mature zygotic embryos on semi-solid M1 medium. After initiation, PEMs were subcultured on semi-solid M2 medium for proliferation (Figure 3A). Somatic embryos developed following transfer of PEMs from M2 medium to semi-solid M4 medium. The PEMs were capable of producing a large number of somatic embryos over several months. However, the initiation of somatic embryos in this system was obviously not synchronized. Since highly efficient polyploid induction relies on the synchronization of the tissue culture process, efforts were made to design a system in which a high frequency of somatic embryogenesis occurred synchronously. The PEMs contained a heterogeneous population of cell aggregates of different sizes and morphologies. For synchronization of somatic embryogenesis, it was important that the cell aggregates are homogeneous in terms of cell cluster size and morphology. After grinding and dispersion of the PEMs with a magnetic agitator in liquid M3 medium, ECAs with a diameter between 100 and 200 μm (Figures 3B,C) were obtained by sieving with nylon screens with 100 and 200 μm pores. ECAs with a diameter between 100 and 200 μm were pipetted onto filter paper and cultured along with the filter paper on semi-solid M4 medium. Globular embryos began to appear in the second week, and there was a rapid increase in the number of somatic embryos by the third week. The somatic embryo differentiation process largely ceased after 4 weeks of culture (Figures 3D,E). ECAs with a diameter of 100–200 μm were the smallest cell aggregates in which embryogenesis could be efficiently induced. It was estimated that 100 ECAs produced an average of 48 somatic embryos within 4 weeks culture. This is an effective regeneration system in which somatic embryogenesis occurs efficiently and synchronously. This system was the basis for the subsequent polyploid induction experiments.

### The Regeneration Patterns of Embryogenic Cell Aggregates After Colchicine Treatment

**FIGURE 4:**
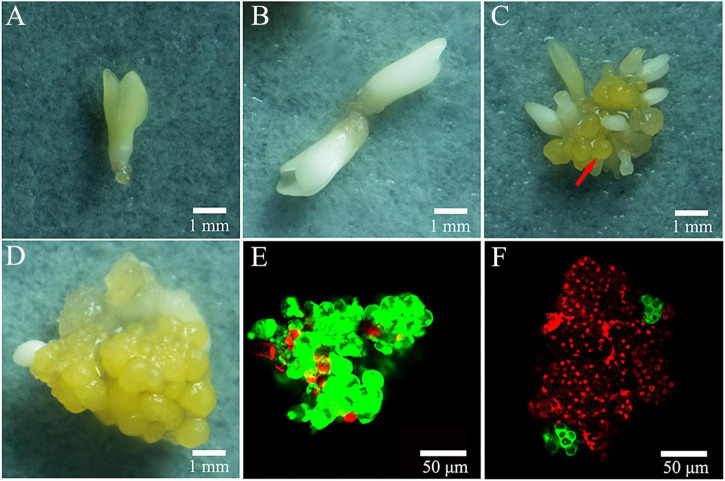
Regeneration patterns of colchicine treated ECAs after 4 weeks regrowth culture. **(A,B)** Somatic embryo differentiation without new ECA formation. **(C)** New ECA (arrow) formation with many somatic embryos forming. **(D)** New ECA formation with scarce somatic embryo differentiation. **(E)** ECA double stained with FDA and PI. **(F)** Colchicine treated ECA (0.2% for 72 h) double stained with FDA and PI following 72 h regrowth culture.

When plating the colchicine treated ECAs onto filter paper and culturing on semi-solid M4 medium, the regeneration process was not obviously delayed when compared to the control treatment without colchicine. Following 4 weeks of culture, the regeneration responses were observed and recorded (Figure 3F). Three types of regeneration patterns could be identified: 1, somatic embryo differentiation without new ECA formation (Figures 4A,B); 2, somatic embryo differentiation with new ECA formation (Figure 4C); and 3, new ECA formation with scarce somatic embryo differentiation (Figure 4D). Although the ECAs showed varying regrowth levels after they had been exposed to different colchicine treatment, these three types of regeneration patterns were observed in all the colchicine treatments. Each regeneration response was initiated from a single colchicine treated ECA which grew into one of the three types of regeneration patterns, and were denominated as single-ECA-derived colonies. When each single-ECA-derived colony was picked up and cultured on semi-solid M4 medium, the majority of them grew into a cluster of somatic embryos and new ECAs. The morphology of the somatic embryos differentiated from single-ECA-derived colonies was normal when compared with the control treatment without colchicine. We did not observe any deformed structures or abnormal morphologies which can be associated with the cytotoxic effects of colchicine. It seems that the use of very small ECAs allowed efficient rinsing of colchicine, thus minimized the carryover effect in the regeneration phase.

Confocal laser scanning microscope has been used to trace the cellular origin of the single-ECA-derived colonies. ECAs with a diameter of 100–200 μm were double stained with FDA and PI immediately after grinding and sieving. Most cells in the ECA emitted green light, indicating their live status (Figure 4E). A few peripheral cells of ECA emitted red light, indicated that they were injured in the grinding and sieving process (Figure 4E). After 72 h of regrowth culture following colchicine treatment (0.2% for 72 h), most of the cells in the colchicine treated ECA were determined to be dead as they emitted red light, evidencing the cytotoxic effect of colchicine (Figure 4F). However, one or two clusters of about 4–8 cells, which stained green appeared on the surface of colchicine-treated ECAs (Figure 4F). It was evident that these cell clusters were formed by the division of one or a very limited number of periphery cells that survived the colchicine treatment. These cell clusters grew into different types of single-ECA-derived colonies following subsequent culture.

### The Regrowth Rate and Embryogenic Potential of Embryogenic Cell Aggregates After Colchicine Treatment

**FIGURE 5:**
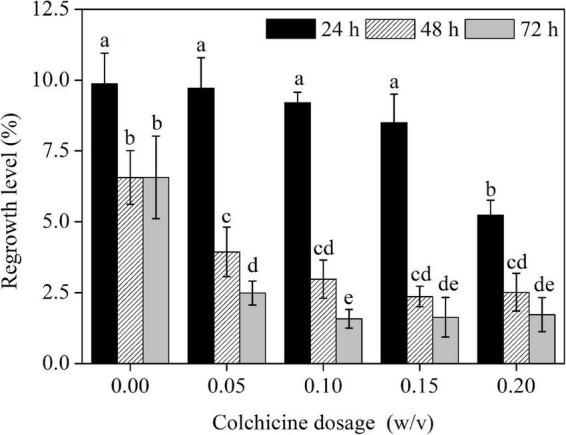
The effects of colchicine treatments on the regrowth levels of ECAs of *Magnolia officinalis*. Data are mean ± SD of three replicates. Means with different letters are significantly different (*p* < 0.05).

The regrowth rate of ECAs following various treatments were quantified after 4 weeks of regrowth culture. The control ECAs without colchicine treatment but suspended in liquid M3 medium for 24 h had a regrowth rate of 9.9% (Figure 5). This response was lower than that achieved with ECAs plated on a piece of filter paper and cultured on semi-solid M4 medium immediately after grinding and sieving (data not shown). This indicated that suspension in liquid M3 medium alone could negatively affect the regrowth ability. The regrowth rates decreased when ECAs were suspended in liquid M3 medium for 48 and 72 h, but this result was not statistically significant. Colchicine treatments at four concentrations (0.05, 0.1, 0.15, and 0.2%) for 24 h caused a slight decrease in regrowth rates compared with control ECAs without colchicine treatment but suspended in liquid M3 medium for 24 h. However, colchicine treatments at these four concentrations for 48 and 72 h led to significant reductions in regrowth when compared with colchicine treatment for 24 h and the control treatment without colchicine. Overall, the regrowth rate was negatively correlated with concentration and duration of colchicine treatments.

The embryogenic potential was determined as the average number of somatic embryos produced per ECA following 4 weeks of regrowth culture. Control and colchicine-treated ECAs produced an average of 2.2–3.7 somatic embryos, which was not statistically different (Supplementary Table S1). This indicates that the embryogenic potential of ECAs was not affected by the colchicine treatment.

### Polyploid Analysis

The somatic embryos derived from colchicine treated ECAs germinated normally and converted into plantlets in glass culture vessels containing 100 mL of semi-solid M4 medium. Flow cytometry analysis was used to determine the ploidy level of the somatic embryo derived plantlets. With increasing concentration and duration of colchicine treatment, the number of somatic embryos and plantlets recovered decreased due to the decreased regrowth rate of ECAs. The strongest colchicine treatment in the present study (i.e., 0.2% for 72 h), yielded 24 *in vitro* plantlets, which is the minimum number among other treatments. Therefore, twenty-four plantlets have been randomly selected from the control and each colchicine treatment for flow cytometry analysis. No polyploids were detected in the control without colchicine treatment (Table 2). The frequency of tetraploids increased with increasing colchicine concentration and exposure time. All the plantlets (100%) regenerated from 0.2% colchicine treatment for 72 h were tetraploid (Table 2). Fifteen (62.6%) tetraploid plantlets were identified from the 0.15% colchicine treatment for 72 h (Table 2). Relatively high levels of tetraploid plantlets were also identified from ECAs treated with 0.15 and 0.2% colchicine for 48 h (Table 2). One plantlet (4.2%) showing an intermediate cytotype was detected in three colchicine treatments (0.05% for 72 h, 0.15% for 72 h, and 0.2% for 48 h). Mixoploid plantlets were not detected in the present study.

**TABLE 2:** The ploidy level of *in vitro* plantlets regenerated from colchicine treated ECAs of *Magnolia officinalis* by somatic embryogenesis.

Colchicine concentration (% w/v)	Duration (h)	No. of diploids (%)	No. of plants with intermediate cytotype (%)	No. of tetraploids (%)
0.00	24	24 (100%)	0	0
	48	24 (100%)	0	0
	72	24 (100%)	0	0
0.05	24	24 (100%)	0	0
	48	24 (100%)	0	0
	72	22 (91.7%)	1 (4.2%)	1 (4.2%)
0.1	24	24 (100%)	0	0
	48	23 (95.8%)	0	1 (4.2%)
	72	22 (91.7%)	0	2 (8.3%)
0.15	24	24 (100%)	0	0
	48	19 (79.2%)	0	5 (20.9%)
	72	8 (33.3%)	1 (4.2%)	15 (62.6%)
0.2	24	23 (95.8%)	0	1 (4.2%)
	48	15 (62.5%)	1 (4.2%)	8 (33.4%)
	72	0	0	24 (100%)

### The Establishment and Verification of Homogeneous Tetraploid Cell Lines

When colchicine treated ECAs were plated onto a piece of filter paper and cultured on semi- solid M4 medium, each surviving ECAs regenerated into a single-ECA-derived colony. Three types of regeneration response could be distinguished in single-ECA-derived colonies (Figure 4). Each single-ECA-derived colony was picked up with the help of a stereomicroscope and then transferred to semi-solid M4 medium without filter paper for further culture. The majority of them grew into a cluster of somatic embryos and callus, irrespective of their initial composition. Even somatic embryos without callus formation (Figures 4A,B) could form some callus tissue at the position of the radicle after 4 weeks of culture on semi-solid M4 medium. Somatic embryos from each single-ECA-derived colony were converted into plantlets and their ploidy levels were determined.

**FIGURE 6:**
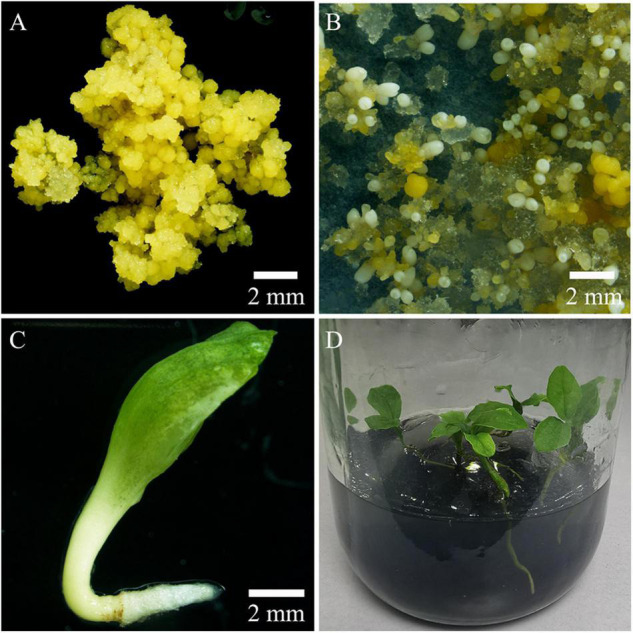
Production of tetraploid plantlets by somatic embryogenesis. **(A)** Tetraploid embryogenic cell line of *Magnolia officinalis*. **(B)** Somatic embryo differentiation from tetraploid ECAs plated on a piece of filter paper and cultured on M4 medium for 6 weeks. **(C)** Tetraploid somatic embryo germinating on M4 medium. **(D)** Conversion of tetraploid somatic embryos into plantlets.

**FIGURE 7:**
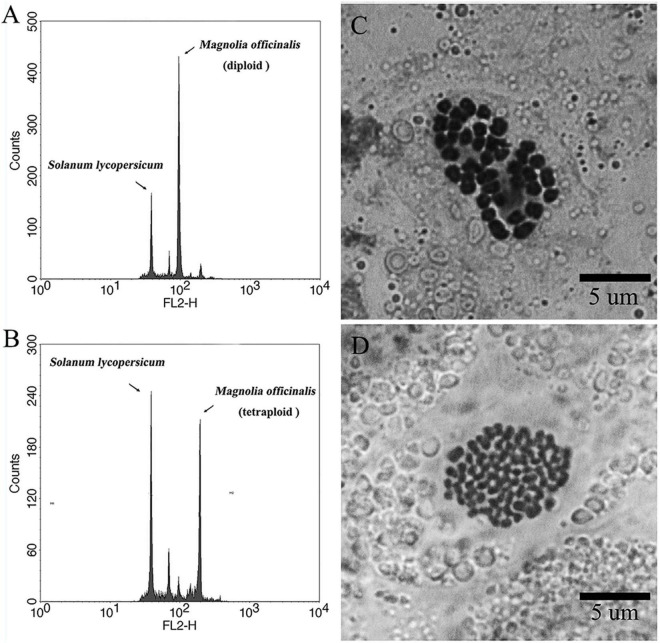
Representative flow cytometry histograms and karyotypes of diploid and tetraploid *Magnolia officinalis*. Representative flow cytometry histograms of the nuclear DNA content in **(A)** diploid and **(B)** tetraploid leaf samples of *M. officinalis*. Chromosomes prepared from **(C)** diploid (2*n* = 2× = 38) and **(D)** tetraploid (2*n* = 4× = 76) samples of *M. officinalis*.

In the present study, high levels of tetraploid induction were obtained from several colchicine treatments. All the plantlets (*n* = 24, 100%) recovered from the treatment of ECAs with 0.2% colchicine for 72 h were determined to be tetraploid (Table 2). It was also established that a very limited number of cells survived colchicine treatment at 0.2% for 72 h (Figure 4F). These surviving cells were obviously the common cellular origin of the somatic embryos, as well as the callus tissues in the single-ECA-derived colonies. On the basis of shared cellular origin, it was assumed that tetraploid ECAs might exist in the single-ECA-derived colonies from which tetraploid somatic embryos derived plantlets were identified at high frequency. When each granular ECA (Figure 4C arrow) from colonies with a high percentage of tetraploid plantlets were carefully picked up under a stereomicroscope and cultured on semi-solid M2 medium, they proliferated into PEMs with a diameter of about 1 cm within 3 months. These were denominated as a cell line. By this method, 23 cell lines were established from single-ECA-derived colonies derived from colchicine treatment at 0.2% for 72 h. Flow cytometry analysis revealed that all 23 cell lines were tetraploid, and no mixoploid tissues were detected. Somatic embryos were successfully differentiated from these tetraploid cell lines and germinated into plantlets (Figure 6). All the somatic embryos and plantlets derived from tetraploid cell lines were determined to be tetraploid by flow cytometry (Figures 7A,B), indicating that ploidy stability was maintained through the somatic embryogenesis process. The ploidy level of plantlets derived from tetraploid cell lines were further confirmed by chromosome counting (Figures 7C,D). The diploid plantlets showed 2*n* = 38 chromosomes (Figure 7C) compared with 2*n* = 76 in the tetraploid plantlets (Figure 7D).

### Comparison of Somatic Embryogenesis Processes of Diploid and Tetraploid Cell Lines

The establishment of tetraploid cell lines allowed comparison to be made of somatic embryogenesis processes of tetraploid and diploid cell lines of *M. officinalis*. One representative tetraploid cell line was selected for somatic embryo induction and comparison with the original diploid cell line. Tetraploid ECAs with a diameter of 200–450 μm were found to produce the most somatic embryos when compared with other size ranges of tetraploid ECAs, and this size range was used for the comparison following grinding and sieving of PEMs. These diploid and tetraploid ECAs were then pipetted onto a filter paper and cultured along with filter paper on semi-solid M4 medium in the same time. Globular somatic embryos started to appear in both diploid and tetraploid cell lines after 3 weeks’ culture. However, the further differentiation and maturation of tetraploid somatic embryos lagged behind the diploid. Cotyledonary somatic embryos were observed at the 4th week in the diploid line. A few diploid somatic embryos even germinated precociously by the 5th week. In contrast, tetraploid somatic embryos were observed rarely by the 6th week and the majority of the somatic embryos were still in the globular stage at this time of culture. Apart from requiring extended incubation on semi-solid M4 medium, the further maturation of the tetraploid somatic embryos was normal.

**FIGURE 8:**
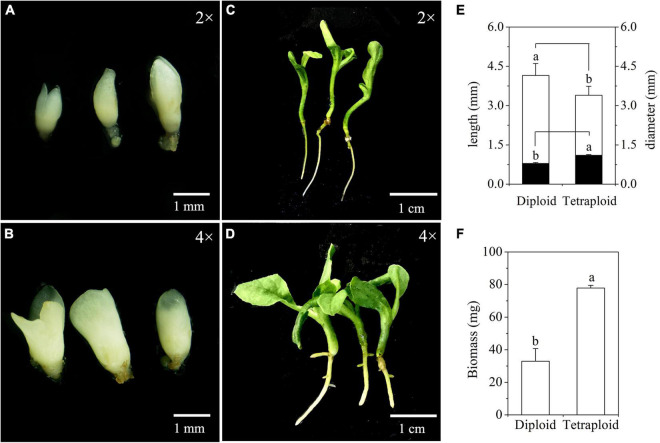
Characterization of somatic embryos and *in vitro* plantlets derived from diploid and tetraploid embryogenic cells of *Magnolia officinalis*. Diploid **(A)** and tetraploid **(B)** somatic embryos 5 weeks after somatic embryo initiation. Diploid **(C)** and tetraploid **(D)** plantlets conversion from somatic embryos. **(E)** The length (open bars) and diameter (closed bars) of diploid and tetraploid somatic embryos of *M. officinalis* 6 weeks after somatic embryo initiation (*n* = 30). **(F)** The biomass of diploid and tetraploid plantlets of *M. officinalis* 3 months after somatic embryo germination (*n* = 12).

Differences in embryogenic potential between diploid and tetraploid cell line of *M. officinalis* were observed. A total of 73 somatic embryos were produced by culturing 50 tetraploid ECAs with a diameter of 200–450 μm (five plates with 10 ECAs), which represented the highest embryogenic potential among other size ranges of tetraploid ECAs. However, this response was still much lower than their diploid counterpart, which produced 217 somatic embryos in 50 ECAs with a diameter of 200–450 μm in the same period. Significant differences in the morphologies of diploid (Figure 8A) and tetraploid (Figure 8B) somatic embryos were also observed. The average length and diameter of cotyledonary somatic embryos of tetraploid lines was significantly shorter and wider, respectively, than those of diploid lines (Figure 8E). Overall, tetraploid somatic embryos were shorter and thicker than diploid somatic embryos. This morphology difference makes it is easy to distinguish tetraploid from diploid somatic embryos *in vitro*.

### Tetraploid and Diploid Phenotypes

**FIGURE 9:**
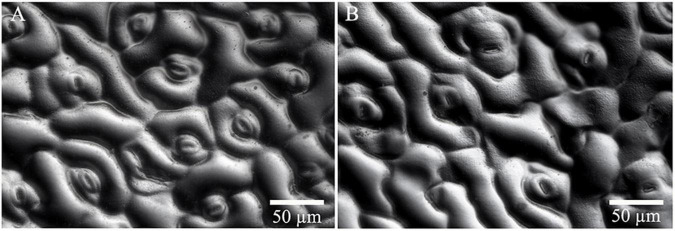
Stomata characteristics of diploid and tetraploid leaves of *Magnolia officinalis*. Nail polish impressions showing stomata on the abaxial surface of diploid **(A)** and tetraploid **(B)** leaves.

Significant differences in morphologies between diploid and tetraploid were observed when somatic embryos converted into *in vitro* plantlets (Figures 8C,D). *In vitro* plantlets of tetraploid lines (Figure 8D) were evidently much larger than those derived from diploid lines (Figure 8C) in the same incubation period 3 months after somatic embryo germination. Compared to diploid line plantlets, the average fresh weight of tetraploid plantlets was increased by 136.3% (Figure 8F). The shoot base and root of tetraploid plantlets (Figure 8D) were also stronger than diploid plantlets (Figure 8C), although root elongation of tetraploid plantlets was delayed. Lateral root initiation was efficient in tetraploid plantlets (Figure 8D), but was not observed in diploid plantlets (Figure 8C). The nail polish imprints showed that the adaxial leave surface stomata of tetraploid plantlets were about 30% longer than diploid plantlets (Figure 9 and Table 3). However, the stomatal density of tetraploid plantlet leaves declined to about 57% of that recorded for diploid plant leaves (Figure 9 and Table 3).

**TABLE 3:** Stomata size (*n* = 24) and density (*n* = 8) of diploid and tetraploid leaves of *Magnolia officinalis*.

	Ploidy level	
	Diploid	Tetraploid
Stomatal density (No./mm^2^)	151.2 ± 5.9^a^	86.3 ± 3.7^a^
Stoma length (μm)	20.90.3^b^	29.8 ± 0.3^a^
Stoma width (μm)	5.4 ± 0.1^b^	6.6 ± 0.1^a^

*Means with different letters are significantly different (Student’s t-test, p < 0.05).*

## Discussion

Here, a novel procedure for the induction and establishment of homogenous tetraploid embryogenic cell lines of the species *M. officinalis* has been described. These cell lines were established from tissues treated with antimitotic agents by manipulation of the regeneration processes. The ploidy level of the synthetic tetraploid cell lines was confirmed by flow cytometry. The new tetraploid cell lines were capable of producing somatic embryos at high frequency. These somatic embryos were successfully germinated into plantlets and their ploidy level confirmed to be tetraploid by flow cytometry and chromosome counting, indicating ploidy stability of the new polyploid cell lines through the somatic embryogenesis process. Through a process of scaling-up, polyploid cell lines could provide substantial numbers of polyploid plantlets for further phenotypic evaluation and for use in breeding programs.

In the present study, several tetraploid embryogenic cell lines were successfully established and confirmed from single-ECA-derived colonies following 0.2% colchicine treatment for 72 h, which produced 100% tetraploid somatic embryos. Previous polyploid induction protocols have been designed to maximize the number of cells affected by the antimitotic agent, increase polyploid production and reduce cytochimera formation (Dhooghe et al., 2011; Touchell et al., 2020). Our results, however, suggest that regeneration from a minimal number of cells could greatly facilitate the establishment of homogeneous polyploid cell lines. Considering the high frequency production of tetraploid somatic embryos and tetraploid cell lines, it was important to confirm the cellular origin of the single-ECA-derived colonies, which were determined using histological methods. FDA and PI double staining of ECAs treated with 0.2% colchicine for 72 h and recovered for another 72 h revealed that most cells in the ECA died, while only a few of periphery cells survived and regenerated into a cluster of somatic embryos and new granular ECAs. Since somatic embryos and new granular ECAs in the specific single-ECA-derived colonies share common and limited cellular origin, it is reasonable to assume that they potentially share a common ploidy level.

In the present study, ECAs with a diameter of 100–200 μm, which possess a high potential of proliferation and differentiation, is the key to success. ECAs with a diameter of 100–200 μm were obtained by dispersion and sieving from the initial heterogeneous embryogenic cell lines. Fractionation of size of cell clusters by sieving has been shown to synchronize somatic embryogenesis (Fujimura and Komamine, 1979; Osuga and Komamine, 1994; Kamo et al., 2004; Souza et al., 2011; Awada et al., 2020), and potentially maximizes the number of cells responsive to the antimitotic agents could facilitate greater polyploid production (Dhooghe et al., 2011). ECAs with a diameter of 100–200 μm composed of dozens of cells were the smallest cell aggregates in which somatic embryogenesis could be induced efficiently. The small and uniform size of ECAs not only permits quick and uniform permeation of antimitotic agents, but also allows efficient rinsing of antimitotic agents before regrowth culture. Therefore, the negative carryover effects of colchicine on the subsequent regrowth and differentiation were minimized, limiting the kind of abnormal growth and morphology previously reported (Zeng et al., 2006; Dutt et al., 2010; Parsons et al., 2019). The production of malformed somatic embryos during regrowth or any interruption to the process of the somatic embryogenesis were not observed in the present study, indicating that the procedure for the rinsing out of colchicine was effective. Antimitotic agent exposure can result in reduced and/or loss of embryogenic potential (Wu and Mooney, 2002; Zeng et al., 2006). However, the embryogenic potential of ECAs was not affected by the colchicine treatment when compared with control treatment in the present study. This may also be attributed to the absence of any carryover effect of colchicine during regrowth.

High levels of solid tetraploid induction without mixoploid formation were obtained in the present study. The most efficient treatment was 0.2% colchicine for 72 h, which produced 100% tetraploid plantlets in the regeneration process (Table 2). Colchicine treatment at 0.15% for 72 h and 0.2% for 48 h produced 62.6 and 33.4% tetraploid plantlets, respectively (Table 2). The development of efficient regeneration systems can facilitate an increased production of homogeneous polyploids and reduction of cytochimeras (Touchell et al., 2020). Highly efficient tetraploid plant induction has been achieved by colchicine treatment of embryogenic suspension cultures in grapevine *Vitis vinifera* cv. Mencía, with the most effective colchicine treatment generating 25% tetraploid plantlets (Acanda et al., 2015). Chromosome set doubling using cellular aggregate suspensions *via* an indirect somatic embryogenesis pathway produced 34.9% autotetraploid plantlets for *Coffea canephora* and 21.1% auto-alloctaploid plantlets for *Coffea arabica* (Venial et al., 2020). Mixoploid formation were neither found in our study with *M. officinalis*, nor in *Vitis vinifera* (Acanda et al., 2015) and *Coffea* species (Sanglard et al., 2017; Venial et al., 2020), which highlights the value of regeneration through somatic embryogenesis from a single cell origin to reduce or avoid the production of mixoploids (Acanda et al., 2015; Touchell et al., 2020; Venial et al., 2020). Conversely, all initial cells within the histogenic layers of the meristems need to be affected by the antimitotic agents to produce homogeneous polyploids using nodal segments and shoot apices; otherwise, mixoploid formation occurs (Dhooghe et al., 2011; Touchell et al., 2020). As a measure of how difficult this is, polyploid induction using multicellular meristems usually leads to a high frequency of mixoploids (Roux et al., 2001; Allum et al., 2007; Fernando et al., 2019). There is also the potential problem of reversion of putative polyploids to diploids and/or mixoploids (Allum et al., 2007; Silva et al., 2019). Overall, the present study has demonstrated how the development of an efficient regeneration system improves the production of homogeneous polyploids and reduces cytochimeras.

The manipulation of differentiation and proliferation of colchicine-treated ECAs of *M. officinalis* by the presence or absence of filter paper on semisolid M4 medium is key for the success of the method presented. Filter paper was used during the first phase of regrowth culture for somatic embryo differentiation. The use of filter paper or semi-permeable cellulose acetate membranes has been shown to enhance the induction and proper maturation and conversion of somatic embryos (Merkle et al., 1990; Niedz et al., 2002; Dutt et al., 2010). Alteration of water potential by filter paper or cellulose acetate membranes can dramatically alter the regrowth and differentiation of ECAs (Merkle et al., 1990; Niedz et al., 2002). When single-ECA-derived colonies were transferred onto semi-solid M4 medium without filter paper, the proliferation of new granular ECAs was encouraged and germination of the somatic embryos that had initially formed on the filter paper facilitated.

In the present study, somatic embryos regenerated from 0.2% colchicine treatment for 72 h could be clearly discriminated from those of control, as they are much bigger in size. This kind of “giant” somatic embryos was also observed in other colchicine regimes. When these somatic embryos were selected based on morphology characteristics and the ploidy levels were analyzed by flow cytometry, all were found to be tetraploid. After the tetraploid cell lines were established and verified, somatic embryos were produced and compared with the diploid lines. Generally, tetraploid somatic embryos were shorter and thicker in appearance than those of diploid lines. These findings suggest that the morphology of somatic embryos may be employed as a potential morphology indicator to predict ploidy level to expedite identification of putative tetraploid regenerates at an early stage before plantlet conversion. Other systems show a similar change in morphological phenotype with polyploidization. The *in vivo* autopolyploid induction system in the Chinese jujube tree (*Ziziphus jujuba* Mill.) was established by integrates *in vivo* bud regeneration *via* calluses with polyploid induction. At the mature leaf stage, not just octoploids but also tetraploid, diploid and mixoploid plants could be forecasted with a high degree of accuracy by observing the size and shape of shoots and leaves (Shi et al., 2015). A widespread consequence of polyploidy is an increase in cell size. For example, the tetraploid cell size of “Meiwa” kumquat (*Fortunella crassifolia*) is much larger than the diploid, and the two types could be clearly discriminated by size in a mixed population of diploid and tetraploid cells (Zeng et al., 2006).

*Magnolia officinalis* belongs to the Magnoliaceae, which consists of over 300 species in two genera–*Magnolia* L. and *Liriodendron* L (Rivers et al., 2016). Many magnolia species are widely appreciated around the world as ornamental trees due to their attractive flowers and foliage. The first protocol for somatic embryogenesis of *Liriodendron tulipifera* was published nearly 40 years ago (Merkle and Sommer, 1986). Since then, many magnolia species and hybrids have been shown to be amenable to somatic embryogenesis with protocols similar to that of *Liriodendron tulipifera* (Merkle, 1999; Mata-Rosas et al., 2006; Kim et al., 2007; Park et al., 2012). The efficient method developed for the induction and establishment of polyploid embryogenic cell lines for *M. officinalis* in the present study, is based on the manipulation of the somatic embryogenesis process following antimitotic agent treatment. Our method has great potential for the creation of new polyploid germplasm for species with established somatic embryogenesis protocols, especially in the magnolia family.

## Conclusion

Our results with *M. officinalis* demonstrate the potential of establishing polyploid embryogenic cell lines *via* induction along the somatic embryogenesis pathway. The use of very small ECAs composed of dozens of cells is highlighted. Only a few cells in the ECAs survived colchicine treatment and regenerated into single colonies. However, the dual potential of differentiation and proliferation of the ECAs was then manipulated to produce somatic embryos for ploidy analysis and new ECAs for cell line establishment, respectively. The present study shown that a high frequency of homogeneous polyploid cell lines can be established by proliferation of single new granular ECAs picked up from colonies with a high presence of polyploid somatic embryos. Somatic embryos and new ECAs in the same colonies derived from each colchicine treated ECA share a common origin from a very limited numbers of cells. The ploidy level of somatic embryos can reflect the ploidy level of cells that survived antimitotic agent treatment and regenerate. This increases the likelihood of being able to establish and purify polyploid cell lines. Our results provide insight into the potential of obtaining new polyploid embryogenic cell lines *via* polyploid induction with a somatic embryogenesis system.

## Data Availability Statement

The raw data supporting the conclusions of this article will be made available by the authors, without undue reservation.

## Author Contributions

LL conceived and designed the experiments and wrote the manuscript. YG, JM, and QX performed the *in vitro* tissue culture experiments and chromosome set doubling experiments and carried out the phenotypic analysis. YG, JM, and YJ performed the flow cytometry analyses. JC and YG performed the Chromosome counting experiment. LL, WL, and HC revised the manuscript. All authors contributed to the article and approved the submitted version.

## Conflict of Interest

The authors declare that the research was conducted in the absence of any commercial or financial relationships that could be construed as a potential conflict of interest.

## Publisher’s Note

All claims expressed in this article are solely those of the authors and do not necessarily represent those of their affiliated organizations, or those of the publisher, the editors and the reviewers. Any product that may be evaluated in this article, or claim that may be made by its manufacturer, is not guaranteed or endorsed by the publisher.
