# Supplementary material for: Establishing Tetraploid Embryogenic Cell Lines of Magnolia officinalis to Facilitate Tetraploid Plantlet Production and Phenotyping
Source: Front Plant Sci. 2022 May 4;13:900768. doi: 10.3389/fpls.2022.900768 (PMC9115471; doi:10.3389/fpls.2022.900768)
Supplement: Supplementary file 1 [file Table_1.DOCX]

## Table S1 The effect of colchicine on the embryogenic potential of ECAs of *M. officinalis*. The mean number of somatic embryos produced per surviving ECA was determined after four weeks of culture on M4 medium.

| **Colchicine dosage (% w/v)** | **Treatment duration (h)** | **Embryogenic potential** |
| --- | --- | --- |
| **0** | 24 | 3.33±0.246^a^ |
|  | 48 | 2.89±0.22^a^ |
|  | 72 | 3.45±1.23^a^ |
| **0.05** | 24 | 3.01±0.34^a^ |
|  | 48 | 2.85±0.51^a^ |
|  | 72 | 2.66±0.50^a^ |
| **0.1** | 24 | 3.32±0.33^a^ |
|  | 48 | 3.67±0.47^a^ |
|  | 72 | 3.57±1.23^a^ |
| **0.15** | 24 | 3.46±0.44^a^ |
|  | 48 | 2.55±0.39^a^ |
|  | 72 | 2.17±0.70^a^ |
| **0.2** | 24 | 3.03±0.28^a^ |
|  | 48 | 2.25±0.55^a^ |
|  | 72 | 2.29±1.21^a^ |
